# Targeting CXCR4 by a selective peptide antagonist modulates tumor microenvironment and microglia reactivity in a human glioblastoma model

**DOI:** 10.1186/s13046-016-0326-y

**Published:** 2016-03-25

**Authors:** Laura Mercurio, Maria Antonietta Ajmone-Cat, Serena Cecchetti, Alessandro Ricci, Giuseppina Bozzuto, Agnese Molinari, Isabella Manni, Bianca Pollo, Stefania Scala, Giulia Carpinelli, Luisa Minghetti

**Affiliations:** Department of Cell Biology and Neurosciences, Istituto Superiore di Sanità, Rome, Italy; Department of Technology and Health, Istituto Superiore di Sanità, Rome, Italy; Department of Research, Diagnosis and Innovative Technologies, Regina Elena National Cancer Institute, Rome, Italy; Division of Neuropathology, Fondazione IRCCS Istituto Neurologico C. Besta, Milan, Italy; Molecular Immunology, Functional Genomics, Istituto Nazionale per lo Studio e la Cura dei Tumori, “Fondazione G. Pascale” IRCCS, Napoli, Italy

**Keywords:** CXCR4, CXCL12, Glioma, Plerixafor, Microglia, Macrophage polarization, Tumor microenvironment, GAM

## Abstract

**Background:**

The CXCL12/CXCR4 pathway regulates tumor cell proliferation, metastasis, angiogenesis and the tumor-microenvironment cross-talk in several solid tumors, including glioblastoma (GBM), the most common and fatal brain cancer. In the present study, we evaluated the effects of peptide R, a new specific CXCR4 antagonist that we recently developed by a ligand-based approach, in an in vitro and in vivo model of GBM. The well-characterized CXCR4 antagonist Plerixafor was also included in the study.

**Methods:**

The effects of peptide R on CXCR4 expression, cell survival and migration were assessed on the human glioblastoma cell line U87MG exposed to CXCL12, by immunofluorescence and western blotting, MTT assay, flow cytometry and transwell chamber migration assay. Peptide R was then tested in vivo, by using U87MG intracranial xenografts in CD1 nude mice. Peptide R was administered for 23 days since cell implantation and tumor volume was assessed by magnetic resonance imaging (MRI) at 4.7 T. Glioma associated microglia/macrophage (GAMs) polarization (anti-tumor M1 versus pro-tumor M2 phenotypes) and expressions of vascular endothelial growth factor (VEGF) and CD31 were assessed by immunohistochemistry and immunofluorescence.

**Results:**

We found that peptide R impairs the metabolic activity and cell proliferation of human U87MG cells and stably reduces CXCR4 expression and cell migration in response to CXCL12 in vitro. In the orthotopic U87MG model, peptide R reduced tumor cellularity, promoted M1 features of GAMs and astrogliosis, and hindered intra-tumor vasculature.

**Conclusions:**

Our findings suggest that targeting CXCR4 by peptide R might represent a novel therapeutic approach against GBM, and contribute to the rationale to further explore in more complex pre-clinical settings the therapeutic potential of peptide R, alone or in combination with standard therapies of GBM.

**Electronic supplementary material:**

The online version of this article (doi:10.1186/s13046-016-0326-y) contains supplementary material, which is available to authorized users.

## Background

Glioblastoma (GBM), the most frequent and malignant type of glioma, represents the most common and fatal cancer of the central nervous system. Despite advances in neurosurgery, chemotherapy and radiation therapy, the median survival is approximately 15 months, due to the recurrent and infiltrative nature of this tumor [1]. A deep knowledge of GBM biology could lead to targeted therapeutic approaches based on the inhibition of tumor specific proteins or molecular pathways [2].

Gliomas are surrounded by resident non-tumor cells, including infiltrating lymphocytes and a large number of glioma-associated microglia/macrophages (GAMs), which are correlated with the malignancy grade [3, 4] and can support tumor structure, angiogenesis, growth and invasiveness in response to autocrine and paracrine molecular regulators secreted by tumor cells [5, 6]. Metabolic cues and molecular signals from glioma can instruct GAMs to down-regulate the M1 pro-inflammatory potentially anti-tumoral functions and to acquire a M2 anti-inflammatory, immunosuppressive and pro-angiogenic phenotype, able to foster neo-vascularization, matrix remodeling, tumor growth, metastasis and recurrence, as well as to suppress adaptive immunity, as reported for other solid tumors-associated macrophages [7–10]. Although the molecular signals implicated in macrophages-tumor cross talk remain largely unknown, chemokines and their cognate receptors are receiving increasing attention [11–13]. In particular, the chemokine receptor CXCR4, a G protein-coupled receptor initially linked with leukocyte trafficking and with HIV infection [14], is expressed in various tumors including GBM, and high levels of expression have generally negative prognostic significance [15–17]. The binding of its ligand, the chemokine stromal cell-derived factor 1-α (SDF1-α, or CXCL12), triggers different downstream signaling pathways in tumor cells and in cells of the surrounding microenvironment, which result in a variety of cellular responses including angiogenesis, metastasis, proliferation and survival [18–20]. It has been reported that high CXCL12 levels in the tumor may attract CXCR4-positive vascular and inflammatory cells that, once within the tumor, secrete tumor promoting cytokines as well as growth and pro-angiogenic factors [21–24]. In the recent years, multiple preclinical studies in animal models of glioma have demonstrated that disrupting the CXCL12/CXCR4 pathway by specific antagonists affects tumor growth, vasculogenesis and post-radiation recurrence, suggesting that this approach represents a promising strategy for glioblastoma therapy [25–28]. The CXCR4 antagonist Plerixafor is the most studied and clinically advanced compound among the agents that inhibit CXCL12/CXCR4 signaling [29]. It has been reported to inhibit the growth of glioblastoma [25], and to limit the survival of cancer stem cells derived from human glioblastoma [30]. However, Plerixafor lacks CXCR4 specificity because it also binds the other high-affinity receptor for CXCL12, CXCR7, as an allosteric agonist [31]. CXCR7 has been recently identified in several tumors, including GBM in which it is involved in many aspects of tumor biology [32, 33]. In addition, cardiotoxicity and other adverse events were reported following long-term usage of Plerixafor [34], prompting the search for new safer and selective CXCR4 inhibitors suitable as anti-GBM agents.

Recently, we have developed a new family of CXCR4 antagonists by a ligand-based approach [35]. Three of these novel cyclic peptides impaired CXCR4 function in vitro (competition with anti-CXCR4 antibody binding, ligand dependent migration, calcium efflux and P-Erk activation) and in vivo, reducing lung metastases in mice injected with B16-CXCR4 mouse melanoma cells and K7M2 mouse osteosarcoma cells. In the same study we also demonstrated that these peptides do not affect CXCR7 and CXCR3 binding [35].

In the present study, we evaluated, in a GBM model, the potential effects of one of these novel cyclic peptides, peptide R, which exhibited the best efficacy in inhibiting CXCL12-dependent migration, ERK phosphorylation and wound healing in human melanoma cells [35], and strongly affected migration in osteosarcoma cells cultured in presence of bone-marrow derived mesenchymal stem cells [36]. The human glioblastoma U87MG cell line was used for the evaluation of CXCR4 expression, cell proliferation and migration in in vitro assays, as well as for intracranial xenografts in nude mice for in vivo studies. Peptide R effects were compared to those exerted by Plerixafor. We demonstrate that peptide R generates an unfavorable microenvironment for tumor cells by switching GAMs phenotype towards M1 features, and decreasing intra-tumor vasculature. Our results are in support of a therapeutic potential of peptide R in GBM treatment, alone or in combination with standard therapies.

## Methods

### Glioma cell line and treatments

The human U87MG glioblastoma cell line from the American Type Culture Collection (ATCC) was maintained in MEM (Life Technologies), supplemented with 10 % fetal bovine serum (FBS), 2 mM glutamine, 1 mM sodium pyruvate, 100 μg/ml streptomycin and 100 U/ml penicillin (all from Life Technologies).

After 24 hours of culture in complete medium, cells were serum deprived for 16 h in MEM supplemented with 1 % of bovine serum albumin (BSA; Sigma-Aldrich). Afterwards, medium was removed and replaced by fresh medium; cells were stimulated or not with 100 ng/ml CXCL12 (PeproTech) and cultured in absence or in presence of 10 μM Plerixafor (Sigma Aldrich) or with 10 μM of peptide R for 24, 48 and 72 h, on the basis of a previous study [35].

### Western blot analyses

U87MG cells were seeded at the density of 2.5 × 10^5^/ml in T-25 flasks, treated as described above and lysed in RIPA buffer (150 mM NaCl, 50 mM Tris-HCl, pH 7.5, 1 % Nonidet P-40, 0.5 % sodium deoxycholate, 0.1 % SDS), supplemented with protease and phosphatase inhibitor cocktails (Hoffman-La Roche Ltd). Protein concentrations were determined by Bradford’s protein assay (BioRad Laboratories) and 50 μg of total lysates were resolved by SDS-PAGE (10 % polyacrylamide) under reducing conditions and blotted with the polyclonal anti-human CXCR4 antibody (dilution 1:1000) and anti-β-actin monoclonal antibody (dilution 1:2000) (Sigma-Aldrich), followed by goat anti-rabbit IgG (dilution 1:3000) and goat anti-mouse IgG (dilution 1:3000) (BioRad Laboratories) HRP-conjugated secondary antibodies.

### Analysis of cell proliferation

U87MG cells were seeded in 6-well plates at the density of 2 × 10^5^/2 ml. After 24 h of culture in complete medium cells were serum deprived, as described in the previous section, and then the proliferation rate was monitored by counting live and dead cells by Trypan blue exclusion assay 24, 48 and 72 h after stimulation with CXCL12 (100 ng/ml) and treatment with peptide R or Plerixafor. Results represent three independent experiments performed in triplicate.

### MTT assay

U87MG cells were seeded in 96-well plates at the density of 6 × 10^3^ cells in 200 μl/well and treated with CXCL12, Plerixafor or with peptide R, as described in the previous “Treatments” section. MTT (5 μg/ml) was added at each time point (24, 48, 72 h) during the final 2 h of treatment. After removing cell medium, 100 μl DMSO were added and optical densities measured at 595 nm with a LT-4000MS Microplate Reader (Labtech International Ltd). Measurements were made in triplicates from three independent experiments.

### Apoptosis and necrosis analysis

U87MG cells were seeded at the density of 2.5 × 10^5^/ml in T-25 flasks, treated as already described for 24, 48 and 72 h. Apoptosis was evaluated at each time point by measuring phosphatidylserine externalization using Annexin V-biotin (Bender MedSystems) followed by Alexa Fluor® 488-conjugated streptavidin (Life Technologies), and then analyzed by FACSCalibur flow cytometer (BD Biosciences). Necrosis was evaluated with the Propidium Iodide (PI) supravital staining, as previously described [37].

### Transwell chamber migration and invasion assays

Migration of U87MG cells was analyzed by a Transwell chamber assay [38] using 8 μm-pore inserts (BD Biosciences) which stood in 6-well plates (Corning). Cells were seeded in serum free medium (1 × 10^6^ cells/well) in the Transwell chambers either with or without peptide R or Plerixafor and allowed to migrate for 20 h at 37 °C. To stimulate migration either FBS (10 %), as a positive control of migration, or CXCL12, were added to the medium in the well underneath the insert. Quantitative analysis and SEM observations were performed as previously described [39]. Six fields for each condition were examined.

### Generation of human U87MG tumor xenografts and in vivo treatments

CD1 nude mice (6 week-old) (Charles River) were anesthetized with intraperitoneal (i.p.) administration of ketamine hydrochloride (100 mg/kg) and xylazine (10 mg/kg), then stereotactic injection of 25 × 10^3^ U87MG cells, in 7 μl of PBS, was performed into the right caudate nucleus. After glioma cell implantation, mice were randomly assigned to control and to different treatment groups (Plerixafor or peptide R) (*n* = 10 animals per group). The experiment was repeated three times. Drugs were i.p. injected at 1.25 mg/kg for Plerixafor [25] or at 2 mg/kg for peptide R in sterile PBS [35], starting from the day of cell injection, twice per day, for the entire treatment duration (23 days). Control mice received vehicle alone. In a preliminary set of experiments we assessed the delivery of the compound to the brain by using Peptide R conjugated with a fluorescent tag (Tag-750) and optical imaging (Xenogen IVIS system). The fluorescence was analyzed in vivo and in the dissected brain (Additional file 1: Figure S1A, B, C). All animal procedures were approved conforming to the Italian and European animal welfare laws. Licence: Decreto Ministeriale n. 85/2012-B, 19-03-2012.

### MRI imaging

Magnetic Resonance Imaging (MRI) experiments were performed at 4.7 T, on Agilent-VARIAN INOVA SIS 200/183 system (Varian) equipped with a volume coil as transmitter and a surface coil assembled with a stereotaxic mouse head holder as receiver (RAPID Biomedical). Animals were anesthetized with 1–2 % isofluorane in O_2_ (1 L/min) (Forane, Abbott SpA), and body temperature was maintained constant by means of a water bed at 37 °C. MR images were performed, post subcutaneous (s.c.) administration of Gd-DTPA, with T1-weighted multi-slice (TR/TE = 600/18 ms; NS = 4; slices thickness 0.6–1.0 mm). Tumor volume was evaluated by using a dedicated image browser program (Varian). Mice (*n* = 10 mice per group, for each of the three independent experiments) were subjected to MRI analysis at day 10, 15 and 23 days after cell implantation. An example of T1-weighted multi-slices is given in Additional file 1: Figure S1D.

### Immunofluorescence analysis and Immunohistochemistry

25 × 10^3^ U87MG cells were seeded in 24-well cluster plates onto 12-mm cover glasses. Cells were treated as reported in “Glioma cell line and treatments” section. To selectively detect the receptor expression cells were stained, prior or post-fixation/permeabilization, with the monoclonal anti-human CXCR4 antibody (dilution 1:100, R&D Systems), followed by goat anti-mouse Alexa Fluor®488 (dilution 1:200, Life Technologies). Coverslips were mounted with Vectashield® antifade mounting medium containing DAPI (Vector Laboratories). The observations were performed by confocal laser scanning microscopy (CLSM) on a Leica TCS SP2 AOBS apparatus, using excitation spectral laser lines at 405, 488, 543 and 594 nm. Image acquisition and processing were conducted by using the Leica Confocal Software (Leica Microsystems). Cells stained only with the fluorochrome-conjugated secondary antibody were used to set up acquisition parameters. Signals from different fluorescent probes were taken in sequential scanning mode. Several fields of view (>200 cells) were analyzed for each labeling condition, and representative results are shown.

For the in vivo studies, 23 days after U87MG implantation, animals were sacrificed and brains were processed for immunofluorescence (IF), histochemistry and immunohistochemistry (IHC) analyses. In particular, mice (*n* = 5 per group) were anesthetized and transcardially perfused with PBS and then with 4 % PFA. Brains were removed and post-fixed for 24 h at 4 °C. For IF, after fixation, mouse brains were dehydrated overnight in PBS with 5 and 30 % sucrose. Serial 30 μm-thick coronal sections were cut using a Reichert-Jung Frigocut cryostat and stored at -20 °C in cryoprotective solution (PBS, 30 % ethylene glycol and 30 % glycerol). IF staining was performed on free-floating tumor-bearing sections, as previously described [40]. The following primary antibodies were used in overnight incubations: rat anti-mouse CD11b (1:100, Serotec), rabbit anti-human CD68 (1:200, able to detect mouse, rat and human CD68, Santa Cruz), mouse anti-mouse Arg-1 (1:200, BD Biosciences), rabbit anti-mouse anti-iNOS (1:2000, Millipore), goat anti-human vimentin (1:40, R&D Systems), rabbit anti-mouse/human glial fibrillary acidic protein, GFAP (1:200, Dako), rat anti-mouse CD31 (1:200, Serotec), mouse anti-human/mouse vascular endothelial growth factor, VEGF (1:50, R&D Systems). Sections were then incubated with appropriate biotinylated (Vector Laboratories) or Cy3-conjugated (Jackson ImmunoResearch) secondary antibodies (1:200) for 2 h at room temperature, followed by additional 2 h with Alexa Fluor® 488-conjugated streptavidin (1:200) and DAPI (300 nM in PBS; Sigma-Aldrich), mounted with DABCO medium (Sigma-Aldrich). Images were captured by a DFC420C camera on a Leica DM4000B fluorescence microscope and by a Leica TCS SP2 confocal microscope, as described above. CLSM images were obtained by Z-projection of 25–30 optical sections taken from the bottom to the edge of the tissue sections.

Quantification of fluorescence of CD68, CD11b and iNOS immunoreactivity and colocalization analysis of CD11b and iNOS immunoreactivity were performed using ImageJ software (available at http://rsbweb.nih.gov/ij/), and given as mean values ± SD of fluorescence intensity and of % area of colocalization, respectively.

For histological and IHC analyses, mouse brains (*n* = 5 per group) were fixed in 4 % PFA coronal sections at 4 different levels were cut, in order to discover the extension of the tumor, then dehydrated, paraffin-embedded and sectioned at 4 μm. For the histological analysis, slides were stained with haematoxylin-eosin standard method. For IHC sections were deparaffinized and rehydrated with standard procedures, incubated with 3 % H_2_O_2_ (Sigma-Aldrich) for 15’ to block endogenous peroxidase activity, then incubated with normal goat serum for 30’ and subsequently with mouse monoclonal anti-vimentin (1:200) and rabbit polyclonal anti-GFAP (1:600). For mouse primary antibody (vimentin) to minimize reactivity of secondary anti-mouse antibody we used DAKO ARK™ (Animal Research Kit), based on avidin-biotin and Peroxidase method. For GFAP staining, sections were incubated with anti-rabbit Envision® peroxidase conjugated as secondary antibody, for 1 h at room temperature. All antibodies were from Dako. Finally, slides were reacted with diaminobenzidine (DAB Substrate Chromogen System, Dako), counterstained with haematoxylin and mounted, observations were performed on randomly selected samples per group, and all brain sections obtained with these procedures were analyzed.

### Statistical analysis

Statistical analyses were performed using GraphPad Prism 3.03 Software (GraphPad Software Inc.). All data were compared by two-tailed unpaired Student t-Test or one-way ANOVA. Differences were considered significant when *P* < 0.05.

## Results

### CXCR4 expression in U87MG cells

**Fig. 1:**
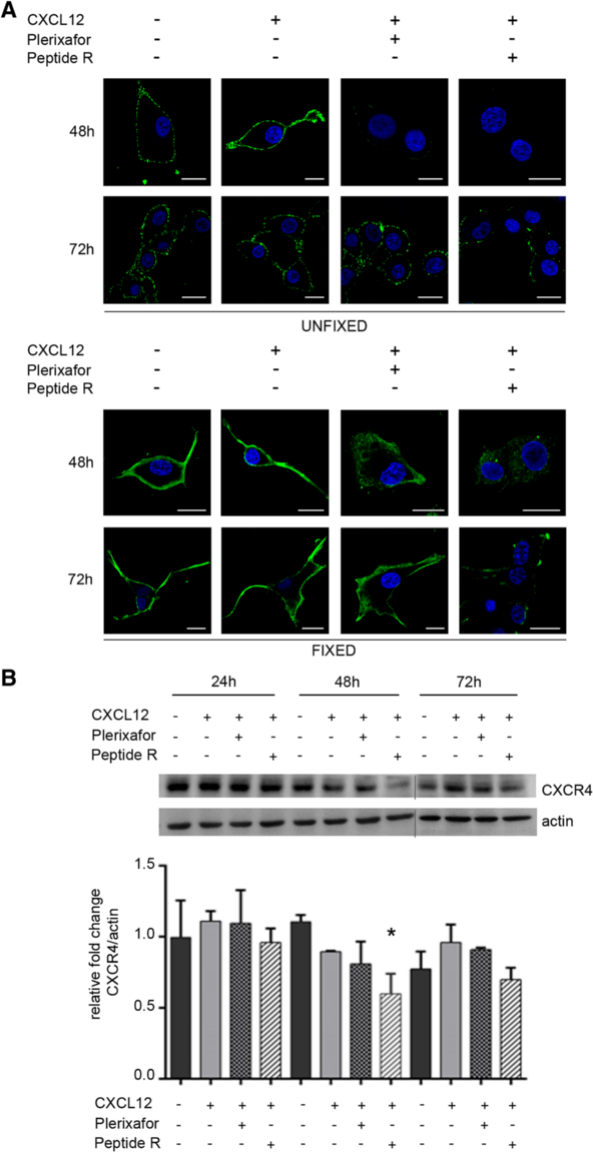
CXCR4 expression in U87MG cell line. **a** CLSM analyses of U87MG cells stimulated (+) or not (-) with CXCL12 (100 ng/ml) and treated (+) with peptide R (10 μM) or Plerixafor (10 μM) or left untreated (−). After 48 or 72 h of culture cells were stained with anti-CXCR4 mAb (green) prior to (UNFIXED, top panel) or after (FIXED, bottom panel) fixation with 3 % PFA. Nuclei were stained with DAPI (*shown in blue*). Scale bars, 15 μm. **b** Representative western blot analysis of CXCR4 detection in U87MG cells processed after 24, 48 and 72 h of treatments as described in (**a**). β-actin was used as loading control. Histograms represent the relative fold change of CXCR4 expression normalized to β-actin, obtained with densitometric analyses of western blot bands (Image J software). Means ± SD of *n* = 3 independent experiments. Statistical analyses were performed with one-way ANOVA, * *P* = 0.0042

Confocal laser scanning microscopy analyses were performed on U87MG cells in order to evaluate CXCR4 expression in response to the different treatments. Cells were first serum deprived (16 h) and stimulated or not with CXCL12 (100 ng/ml). CXCL12-stimulated cells were then cultured in absence or in presence of peptide R (10 μM) or Plerixafor (10 μM) for 24, 48 and 72 h. At each time point, one group of U87MG cultures was stained for CXCR4 detection on cell membrane surface, another one was fixed and permeabilized before staining to assess CXCR4 intracellular expression. No effects on membrane CXCR4 expression were observed after 24 h of treatments (data not shown). At 48 h (Fig. 1a, top panel, Unfixed), CXCL12 stimulation increased CXCR4 membrane expression as compared to unstimulated cells (-CXCL12). The receptor completely disappeared from the cell surface of U87MG cells after peptide R or Plerixafor treatment. After 72 h, CXCR4 down-modulation on cell membrane of peptide R-treated cells was maintained, while in Plerixafor-treated cells the chemokine receptor was re-expressed at levels comparable to unstimulated (-CXCL12) or CXCL12-stimulated cells (+CXCL12). As reported in Fig. 1a (bottom panel, Fixed), after 48 h CXCR4 was still exposed on the membrane upon stimulation with CXCL12 alone; the receptor was instead internalized after treatment with either peptide R or Plerixafor, with the lowest levels of expression detected in the presence of peptide R. At the following time point (72 h), in Plerixafor-treated cells CXCR4 expression and distribution were restored as in control cultures (+CXCL12), while in peptide R-treated cells the reduction of CXCR4 expression was maintained. Densitometric analysis of western blotting experiments showed a significant decrease of CXCR4 total content in U87MG cells exposed to peptide R for 48 h (45 % compared to unstimulated cells and 33 % compared to CXCL12-stimulated cells) and confirmed a downregulation of the receptor after 72 h compared to the other experimental conditions (Fig. 1b).

### Effects of peptide R on U87MG cell growth, viability and migration

**Fig. 2:**
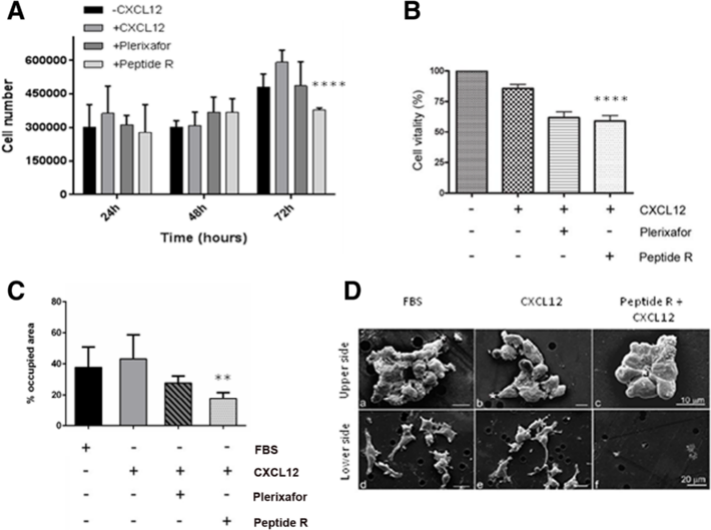
Effects of peptide R on cell proliferation, vitality and migration. **a** Cell counting of U87MG cells stimulated (+CXCL12) or not (-CXCL12) with 100 ng/ml of CXCL12 and treated with Plerixafor (10 μM) or peptide R (10 μM) for 24, 48 and 72 h. Mean values ± SD of *n* = 3 independent experiments. **** *P* = 0.0001, one-way ANOVA, (*asterisks* represented in the figure). *P* = 0.0293 (–CXCL12 cells *versus* peptide R-treated cells), *P* = 0.0005 (+CXCL12 cells *versus* peptide-treated U87MG) (72 h of treatments), unpaired two-tailed Student t-test. **b** MTT assay performed on U87MG cells after 72 h incubation with CXCL12, in the presence of peptide R or Plerixafor, compared to untreated cells (-CXCL12) (means ± SD of *n* = 3 independent experiments). **** *P* = 0.0001 (*asterisks* represented in the figure), one-way ANOVA. *P* = 0.0148 (-CXCL12 cells *versus* Plerixafor-treated U87MG), *P* = 0.0419 (-CXCL12 *versus* peptide R-exposed cells), *P* = 0.0084 (+CXCL12 cells *versus* peptide R-treated U87MG cells), unpaired two-tailed Student t-test. **c** Quantitative analysis of U87MG cells migration (for details see Methods section) in response to 10 % FBS (black bar) or in response to CXCL12 in the absence or presence of peptide R or Plerixafor. The percentages of area occupied by migrating cells are reported as mean values ± SD (*n* = 6). **P = 0.0024 (*asterisks* shown in the figure) one-way ANOVA. *P* = 0.0425 (+CXCL12 *versus* + Plerixafor), *P* = 0.0029 (+CXCL12 *versus* + peptide R cells), unpaired two-tailed Student t-test. **d** Scanning electron microscopy (SEM) observations were performed at the end of the 20-hour assays on cells treated as described in (**c**). Imaging was performed on both the upper (panels **a**–**c**) and lower sides (panels **d**–**f**) of the filter. *Asterisks* represent the migration leader elements. Scale bars are indicated

To investigate the possible functional effects induced by CXCR4 down-modulation, we evaluated cell proliferation after exposure of U87MG cells to peptide R or Plerixafor in the same conditions of the previous experiments. Cells were seeded 24 h before adding the different drugs and cell proliferation rate was determined 24, 48, and 72 h after treatments (Fig. 2a). Consistently with an endogenous production of CXCL12 by U87MG cells [41], we observed an increase in cell number over the 72 h of observation in the absence of added chemokine (Fig. 2a, black bars). Minimal or no changes in cell proliferation were observed at 24 and 48 h of cell culture. At 72 h CXCL12 moderately increased the cell number; after peptide R treatment a 35 % reduction of cell proliferation was observed compared to CXCL12-stimulated cells, and a 20 % reduction compared to unstimulated (-CXCL12) cells. Plerixafor-treated cells showed a moderate reduction in cell proliferation compared to CXCL12-stimulated cells, which did not reach statistical significance.

Evaluation of Annexin V-positive or PI-bright cells at the same time points showed that neither peptide R nor Plerixafor exerted any appreciable apoptotic or necrotic effect (data not shown). Next, cell viability was assessed by MTT assay. No significant effects were induced by both treatments at 24 and 48 h (data not shown); however, a significant reduction in the metabolic activity of U87MG cells was observed in both peptide R- and Plerixafor-treated cells (Fig. 2b).

Since we previously showed that peptide R inhibits cell motility of melanoma and osteosarcoma cells [35, 36], we tested the effects exerted by peptide R and by Plerixafor, on glioma cells migration. U87MG cells, in serum free medium, were seeded in transwell chambers and allowed to migrate towards the chemokine CXCL12 (added to the lower chamber medium) in the absence or presence of peptide R. In addition to CXCL12, FBS was also used as positive control for migration (black bar in Fig. 2c). Quantitative analysis by computer-assisted light microscopy (Fig. 2c), showed that, as expected, CXCL12 stimulated U87MG cell migration, Plerixafor treatment did not induce significant changes, while peptide R significantly reduced the percentage of area occupied by migrating cells. Scanning electron microscopy (SEM) analysis of the upper side of the filter showed that during migration U87MG cells exposed to FBS or CXCL12 move as chains or group of cells (Fig. 2d_a,b_). The presence of peptide R hampered cell migration through the membrane pores, and U87MG cells appeared organized in tight clusters (Fig. 2d_c_). On the lower side of the filter numerous cells were visible in control (FBS) or CXCL12-treated cells (Fig. 2d_d,e_), while in the presence of peptide R very few cells crossing membrane pores were recorded (Fig. 2d_f_).

### In vivo effects of peptide R in the orthotopic U87MG mouse model

**Fig. 3:**
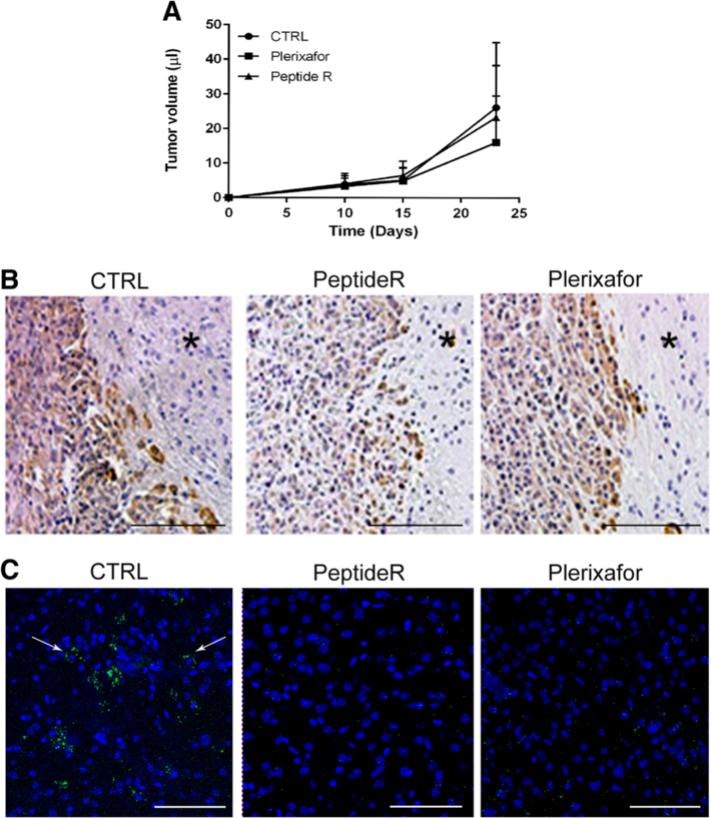
In vivo effects of peptide R treatment of U87MG orthotopic mouse model. **a** Tumor volume measures obtained by MRI analyses performed on mice at day 10, 15 and 23 after U87MG cells implantation and drug administration. *Curves* represent mean value of tumor volumes measures of three independent experiments obtained from mice treated with vehicle (PBS) ● CTRL, ■ Plerixafor and ▲ peptide R (*n* = 10 animals per group, error bars ± SD). **b** Representative brain sections of vehicle-treated (CTRL) or peptide R- or Plerixafor-treated mice stained by immunohistochemistry with the anti-vimentin antibody. Scale bars, 75 μM. **c** Representative CLSM images of Vimentin expression (*green*) of tumor-free contralateral hemispheres of vehicle-treated mice (CTRL), peptide R- and Plerixafor-treated mice. *Arrows* represent Vimentin + cells. Nuclei were stained with DAPI (*blue*). Scale bars 75 μM

The above in vitro results supported the potential anti-tumor effects of peptide R on glioma cells. To test this hypothesis in vivo, we used intracranial orthotopic xenografts of U87MG cells. Tumor volume and localization were evaluated by MRI analyses in mice treated with vehicle, peptide R or Plerixafor, as reported in Methods. Volumetric analysis (Fig. 3a) performed at day 10, 15 and 23 after U87MG cells implantation and treatments did not show any significant change of tumor size in animals treated with both CXCR4 antagonists, compared to control group (PBS-treated mice, CTRL). However, immunohistochemical analyses of brain slices showed a reduction of U87MG cells, as identified by vimentin expression, after treatment with peptide R when compared to Plerixafor or PBS treatment (Fig. 3b), thus suggesting a reduced tumor cell density in peptide R-treated mice. Interestingly, in the contralateral hemisphere no vimentin^+^ cells were detected either after peptide R or Plerixafor treatment (Fig. 3c), suggesting that both CXCR4 antagonists could abrogate dissemination of glioblastoma cells at distant cerebral sites.

**Fig. 4:**
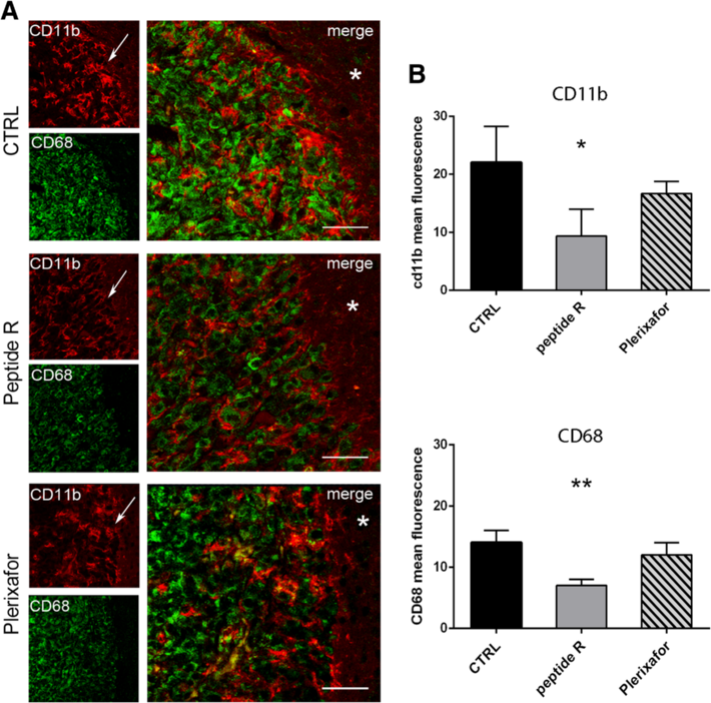
Peptide R effect on macrophages/microglial cells accumulation in U87MG glioma. **a** Representative CLSM analyses of brain sections of vehicle-treated (CTRL), peptide R- or Plerixafor-treated mice stained with anti-CD11b (*red*) and CD68 (*green*, a marker for activated microglia) antibodies. *Arrows* indicate the tumor edge, while *asterisks* (*) indicate the tumor-free parenchyma. Scale bars, 30 μm. **b** Mean intensity of CD11b and CD68 fluorescence were analysed by Image J software and reported as mean values ± SD (*n* = 4–5 images for each treatment group). *P = 0.0420 and **P = 0.0066 (*asterisks* shown in figure), one-way ANOVA. *P* = 0.0476 and *P* = 0.0056 (peptide R *versus* CTRL), unpaired two-tailed Student t-test

To better analyze the in vivo effects of peptide R, we examined the possible involvement of glioma microenvironment in controlling tumor growth and dissemination. By immunofluorescence analysis we investigated the expression of typical markers of GAMs, such as CD11b (cluster of differentiation molecule 11b) and CD68 (a lysosomal glycoprotein used as a marker for activated microglia/macrophages). As shown in Fig. 4, peptide R-treated gliomas showed a reduction of CD11b^+^ and CD68^+^ cells migrated at the tumor edge (Fig. 4a and b) as compared with control and Plerixafor groups. Moreover, in the untreated and Plerixafor-treated tumors we detected more CD11b^+^/CD68^+^ cells respect to peptide R-treated gliomas, suggesting that peptide R treatment perturbs the activation state of GAMs. It is worth noting that glioma cells express the CD68 marker, as confirmed by immunostaining of U87MG cells in vitro (Additional file 2: Figure S2A) and as previously reported [42]. Since the antibody used in the present study recognizes CD68 of mouse and human origin, the observed reduction of CD68 expression could be related not only to GAMs but also to a reduced number of U87MG cells, consistently with the observed decrease in vimentin^+^ cell density (Fig. 3b).

As a marker of astrocyte reactivity, we performed GFAP (glial fibrillary acidic protein) staining by both immunofluorescence and immunohistochemistry. Both techniques evidenced a stronger reactivity in peptide R-treated tumors (Additional file 2: Figure S2B). Since U87MG cells do not express GFAP, the observed staining can be solely associated with astrocytes [43].

### In vivo effects of peptide R on GAM reactivity

In order to better understand the functional relevance of CD11b^+^ cells infiltrating glioma and the influence of CXCR4 inhibition on the functional phenotype of these cells, we analyzed the expression of inducible nitric oxide synthase (iNOS), an enzyme typically involved in microglia/macrophage pro-inflammatory activity (M1 phenotype), and arginase-1 (Arg-1), as a marker of immunosuppressive activity (M2 phenotype).

**Fig. 5:**
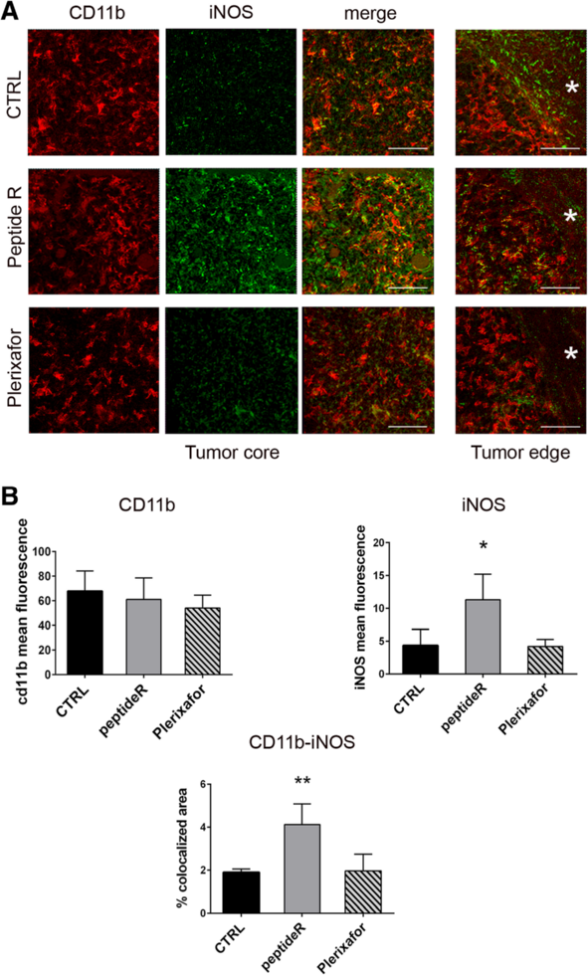
In vivo effects of peptide R treatment on iNOS expression by macrophage/microglia. **a** CLSM analyses of the double staining with CD11b (*red*) and iNOS (*green*) antibodies performed on brain sections of vehicle-treated (CTRL), or peptide R- or Plerixafor-treated mice. Images were taken both in the tumor core and at the tumor edge, as indicated. *Asterisks* (*) indicate the tumor-free parenchyma. Scale bars, 50 μm. **b** Mean intensity of CD11b and iNOS fluorescence (*upper panels*) and percentage of colocalized area (*lower panel*) in the tumor core were analysed by Image J software and reported as mean values ± SD (*n* = 4 images for each treatment group). *P = 0.0226 and **P = 0.0057 (*asterisks* shown in figure), one-way ANOVA. *P* = 0.0448 and *P* = 0.0084 (peptide R *versus* CTRL and Plerixafor), unpaired two-tailed Student t-test

**Fig. 6:**
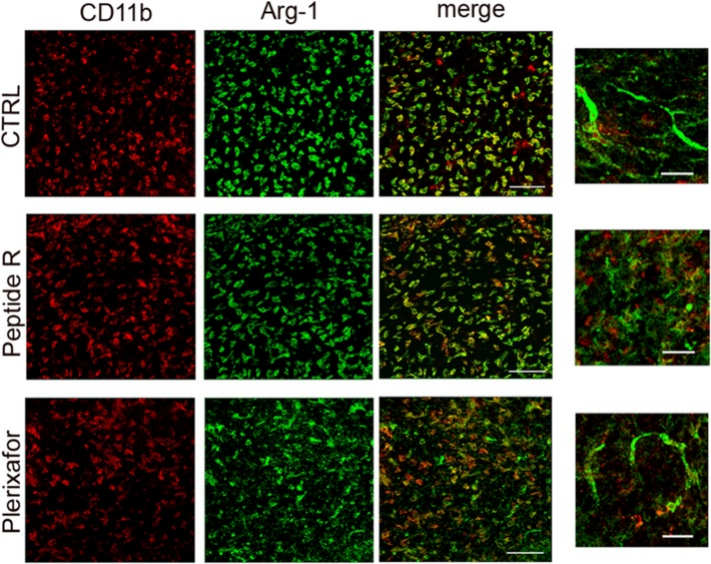
In vivo effects of peptide R treatment on Arg-1 expression by glioma-associated macrophage/microglia. Detection of CD11b- (*red*) and Arg-1- (*green*) positive cells in brain sections of mice treated with peptide R or Plerixafor or left vehicle-treated (CTRL). Scale bars, 75 μm. On the right of each panel a magnification is reported, scale bars 30 μm

**Fig. 7:**
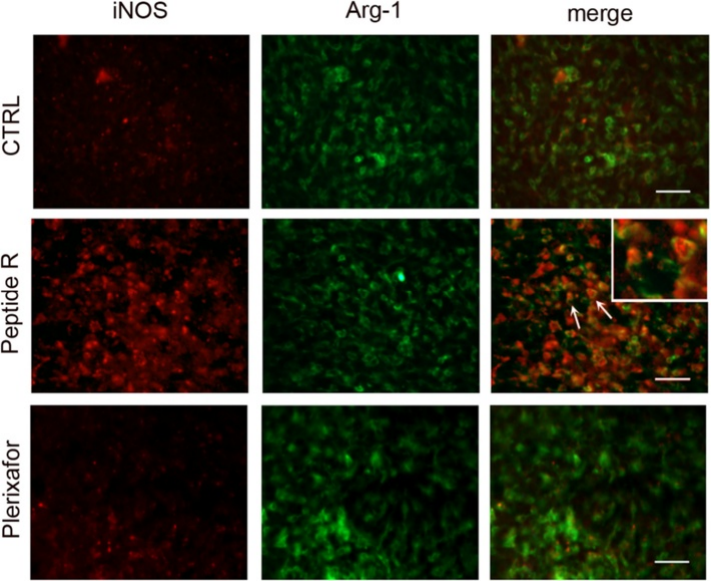
Effects of peptide R on the co-expression of iNOS and Arg-1 enzymes in glioma associated macrophages/microglial cells. Immunofluorescence analyses of iNOS (*red*) and Arg-1 (*green*) performed on brain slices of vehicle-treated (CTRL), peptide R- or Plerixafor-treated mice. The insert in the peptide R-treated sample is a magnification of the area indicated by the *arrows*. Scale bars, 50 μm

Observations performed by double immunofluorescence staining of CD11b and iNOS revealed a strong expression of iNOS by CD11b^+^ cells within the tumors of peptide R-treated mice, compared to control and Plerixafor-treated mice (Fig. 5a and b, Tumor core), suggesting that peptide R treatment promotes M1 properties in GAMs. Analyses of the tumor margin indicated the presence of CD11b^-^/iNOS^+^ cells in the peritumoral area of control gliomas only (Fig. 5a, Tumor edge, indicated by a star). No differences were detected in Arg-1 expression by CD11b^+^ cells among treated and control mice (Fig. 6). However, we detected a higher number of iNOS^+^/Arg-1^+^ in peptide R-treated gliomas (Fig. 7), further supporting the ability of the novel CXCR4 antagonist to modulate the reactivity of GAMs.

Interestingly, untreated and Plerixafor-treated gliomas presented Arg-1^+^ endothelial-like tubular structures in the tumor core. Such structures were absent in peptide R-treated tumors (Fig. 6, higher magnifications on the right).

**Fig. 8:**
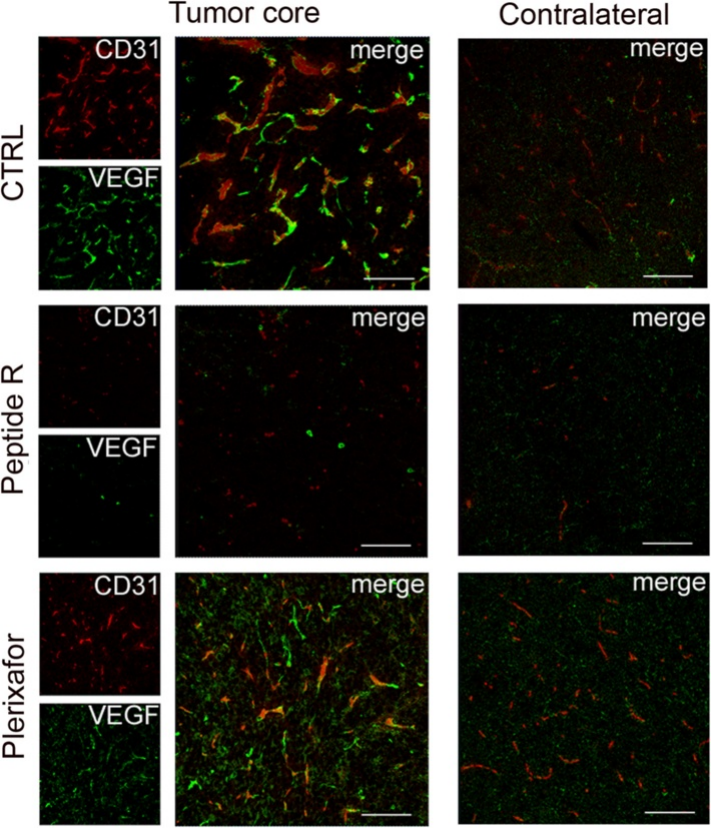
In vivo effects of peptide R on U87MG glioma vascularization. CLSM analyses on brain sections of untreated (CTRL) or peptide R- or Plerixafor-treated mice for the detection of CD31 (endothelial marker, *red*) and VEGF (angiogenic factor, *green*). Images were taken both in the tumor core and in the contralateral hemisphere. Scale bars, 75 μm

The effect of peptide R on tumor vasculature was further investigated by double staining for endothelial related markers, such as CD31 and VEGF. We observed a strong reduction of CD31^+^/VEGF^+^ and CD31^-^/VEGF^+^ cells in the tumor core of mice treated with peptide R, in comparison with control and Plerixafor-treated mice (Fig. 8). In the tumor-free parenchyma, we observed a low expression of VEGF and CD31, without any significant differences among the different treatments (Fig. 8, Contralateral hemisphere).

## Discussion

There is an urgent need in identifying novel therapeutics that will improve survival in GBM patients. The CXCL12-dependent signalling has emerged among the most relevant molecular pathways that can be targeted to successfully interfere with tumor cell proliferation, survival, migration, and radioresistance [18].

Here we show the efficacy of the newly synthesized CXCR4 antagonist peptide R in modulating the intrinsic properties of glioblastoma cells and their microenvironment in an experimental model of GBM. We found that peptide R stably reduced the expression of CXCR4 in human U87MG cells and impaired their metabolic activity, cell proliferation and migration in response to CXCL12 in vitro. Accordingly, in the orthotopic U87MG mouse model, peptide R reduced tumor cellularity, abrogated dissemination of glioblastoma cells at distant cerebral sites, promoted M1 features in GAMs recruited to the tumor area and impaired intra-tumor vasculature.

In our model, peptide R effects were comparable to those observed with Plerixafor, the best-characterized and most widely used antagonist of CXCR4. The two drugs share a common core of ligand-receptor interactions, but they span different subsites possibly explaining differences in specific activities of the two drugs. Indeed, peptide R, unlike Plerixafor, is devoid of agonism to CXCR7, and this property could represent an advantage in the inhibition of CXCL12-dependent signaling [35]. Recent works indicate that glioma and tumor-associated vasculature can express CXCR7, and its expression increases with glioma grade [32]. It is noteworthy that rodent microglia co-express CXCR4 and CXCR7 as a functional receptor unit, which is essential for controlling CXCL12-dependent migration and proliferation, and which is up regulated by classical M1 activators [44].

The entire axis CXCR4–CXCL12–CXCR7 regulates the mammalian target of rapamycin (mTOR) signaling in renal cancer cells, a pathway identified in several human malignancies and effectively blocked by peptide R in these cells [45]. These findings have important implications also for brain tumors, as mTOR is a crucial modulator of inflammatory pathways in microglia and macrophages. Lisi and colleagues [46] reported that mTOR kinase inhibitors polarize glioma-activated microglia to the M1 phenotype. Although the links among the various functions of macrophages, tumor progression and therapy responses are still poorly defined, the M1/M2 balance has emerged as a crucial factor able to control glioma biology and radioresitance [47, 48]. Interestingly, peptide R treatment in the GBM model induced a strong GAM immunoreactivity for the M1 marker iNOS, potentially increasing the anti-tumor killing functions of these macrophages [49]. Although the expression of the M2 marker Arg-1 was not modified in GAMs, the enhanced expression of iNOS indicates that peptide R interferes with the phenotype switching of GAMs towards M2 pro-tumorigenic functions induced by the cross-talk with tumor cells.

Recent results of genome-wide analyses have shown that GAMs represent a unique population, expressing a mixture of M1 and M2-specific genes, although the cellular heterogeneity of GAMs was not investigated [9]. In our model, the co-expression of the two markers (iNOS and Arg-1) in most GAMs of peptide R-treated tumors supports the existence of a mixed phenotype and suggests that peptide R treatment might slow down the polarization to a more homogeneous M2 population. While peptide R treatment induced iNOS expression in GAMs (CD11b positive cells), it conversely reduced iNOS in CD11b negative cells at the tumor margins. The functional significance of this differential regulation of iNOS in different cell populations by peptide R could reflect the complexity of nitric oxide (NO) effects on tumor biology, which appears dependent on NO levels and cellular sources. Low concentrations of NO have been positively associated with tumor growth, migration, invasion, survival, angiogenesis, and metastasis while high NO levels by dendritic/macrophage cells elicit tumoricidal activity [50, 51]. Down-regulation of iNOS in non-macrophage cells at the tumor edges and up-regulation in GAMs in the tumor core could represent a dual protective mechanism of peptide R against tumor growth and dissemination.

In addition, peptide R reduced the presence of CD31/VEGF positive cells and Arg-1-expressing vessel-like structures in the tumor core, suggesting that a reduced aberrant intratumoral vascularization could represent a further important anti-tumoral mechanism of peptide R. Anti-angiogenic therapies in GBM patients indicate the promising benefits of these approaches [52]. This peptide R effect is in line with the recognized role of CXCL12 in angiogenesis and vasculogenesis, but it could also arise by the modulation of GAM reactivity and the retention of M1 functions, less prone to the enhancement of angiogenesis [53–55].

Overall, our data suggest that the potential of peptide R as anti-cancer agent, shown by previous in vivo models of lung metastases (B16-CXCR4 and KTM2 murine osteosarcoma cells) and primary growth of human renal cell xenografts [35] can be exploited also in anti-GBM therapy. Peptide R could enhance the efficacy of standard treatments such as radiation, chemo- and anti-VEGF therapies, known to up-regulate CXCL12 and CXCR4 as part of the escape program that favors tumor recurrence and dissemination at distance, restores the vasculature and promotes GAM recruitment [56, 57]. The recruitment of circulating cells outside of the radiation field that can recolonize and/or stabilize the tumor vasculature after irradiation has been suggested as a mechanism of GBM resistance to irradiation. Consistently, post-radiation therapy inhibition of CXCL12/CXCR4 interaction resulted in the inhibition of tumor recurrence in glioma models [24, 28].

## Conclusions

In our GBM mouse model, Peptide R appears to modulate not only tumor cell intrinsic properties but also those of GAMs and their microenvironment, thus engaging a feed forward loop able to counteract glioblastoma growth and dissemination. These encouraging experimental results, together with data showing no overt toxicity of peptide R in mice [35], contribute to the rationale to further explore the therapeutic potential of peptide R, alone or in combination with standard therapies in more complex pre-clinical GBM models.

## Additional files

Additional file 1: Figure S1. In vivo and ex vivo imaging. In vivo fluorescence images obtained using an IVIS Spectrum bioluminescent and fluorescent imaging system from Xenogen (IVIS Lumina II). Excitation and emission values (640 nm, 700 nm, respectively) were kept constant, and exposure times 7 s were used. A) Example of tumor-bearing mouse at 21th of growth, fluorescence imaging observed at 1 h 30’ after of peptide R (conjugates to VIVOTAG-S 750 fluorochrome) i.v. injected. The animal was placed dorsally under anaesthesia in a light-tight chamber. After final in vivo fluorescence imaging, the animals were euthanized, and the brain of animals were carefully excised, the intensity of fluorescence was increased in brain with tumor (B) respect to the healthy brain (C). Light emitted from the animal/brain appears in pseudocolor scaling. D) Representative multi-slice coronal postcontrast-enhanced (Gd) T1-weighted MR images (4.7 T) of tumor-bearing mouse brain Contrast enhanced-MRI evidences that tumor lesion has almost uniformly disrupted BBB. (TIF 4589 kb) Additional file 2: Figure S2. A) CD68 expression on U87MG cell line. Representative CLSM analysis of CD68 expression (green) on U87MG cells. Nuclei were stained with DAPI (blue). Scale bars, 47 μM. B) In vivo effect of peptide R on astrocytes reactivity. GFAP (marker of astrocyte reactivity) detection by CLSM (on the left) and immunohistochemistry (on the right) on brain sections of mice treated with peptide R or left untreated (CTRL). Asterisks (*) indicate the tumor-free parenchyma. Scale bars, 30 μM. (TIF 1451 kb)

## Abbreviations

GAM: glioma associated macrophages
GBM: glioblastoma multiforme
i.p.: intraperitoneal
MRI: magnetic resonance imaging
s.c.: subcutaneous
