# Supplementary figures and images for: Targeting CXCR4 by a selective peptide antagonist modulates tumor microenvironment and microglia reactivity in a human glioblastoma model
Source: J Exp Clin Cancer Res. 2016 Mar 25;35:55. doi: 10.1186/s13046-016-0326-y (PMC4807593; doi:10.1186/s13046-016-0326-y)

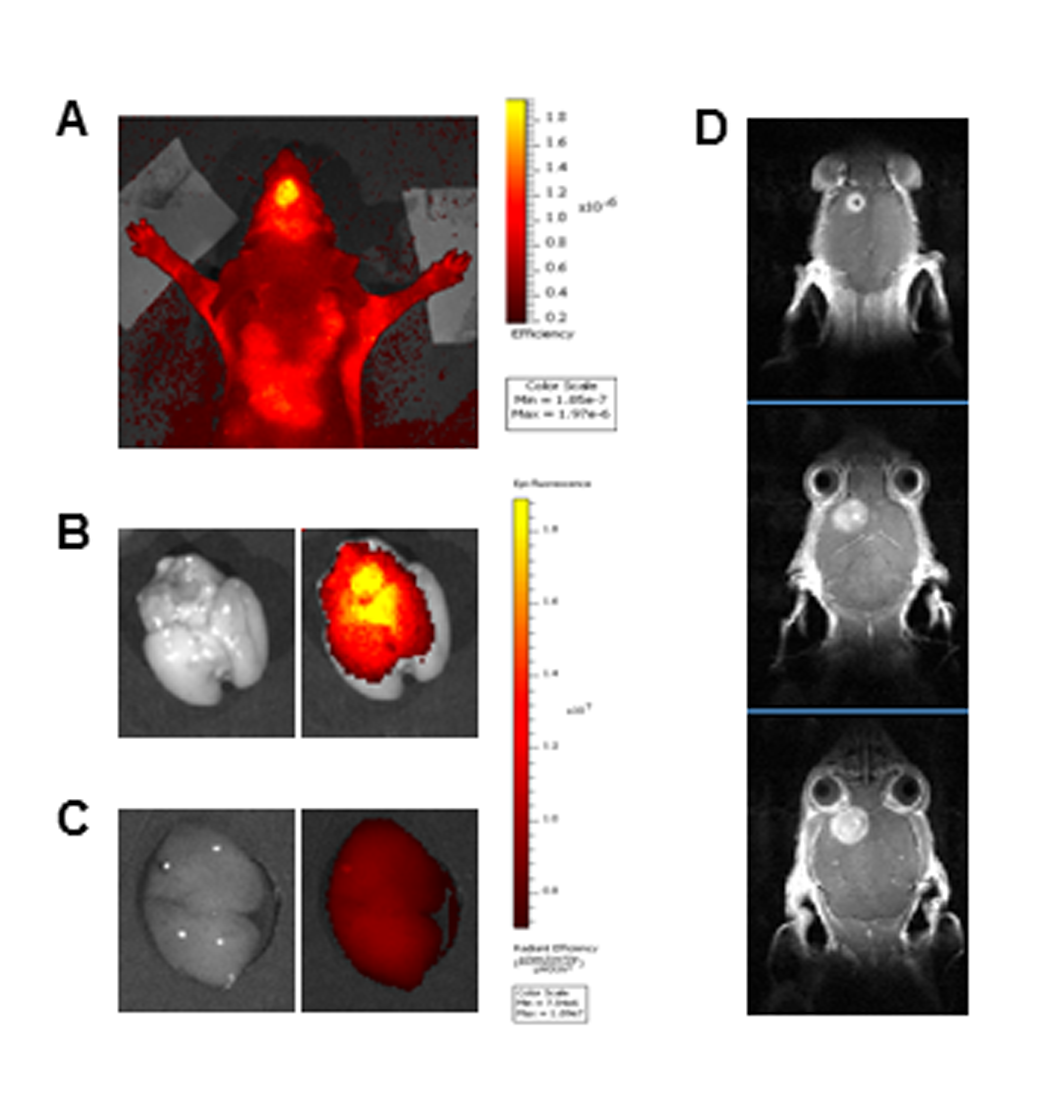

Supplement: Additional file 1: Figure S1. — In vivo and ex vivo imaging. In vivo fluorescence images obtained using an IVIS Spectrum bioluminescent and fluorescent imaging system from Xenogen (IVIS Lumina II). Excitation and emission values (640 nm, 700 nm, respectively) were kept constant, and exposure times 7 s were used. A) Example of tumor-bearing mouse at 21th of growth, fluorescence imaging observed at 1 h 30’ after of peptide R (conjugates to VIVOTAG-S 750 fluorochrome) i.v. injected. The animal was placed dorsally under anaesthesia in a light-tight chamber. After final in vivo fluorescence imaging, the animals were euthanized, and the brain of animals were carefully excised, the intensity of fluorescence was increased in brain with tumor (B) respect to the healthy brain (C). Light emitted from the animal/brain appears in pseudocolor scaling. D) Representative multi-slice coronal postcontrast-enhanced (Gd) T1-weighted MR images (4.7 T) of tumor-bearing mouse brain Contrast enhanced-MRI evidences that tumor lesion has almost uniformly disrupted BBB. (TIF 4589 kb) [file 13046_2016_326_MOESM1_ESM.tif]

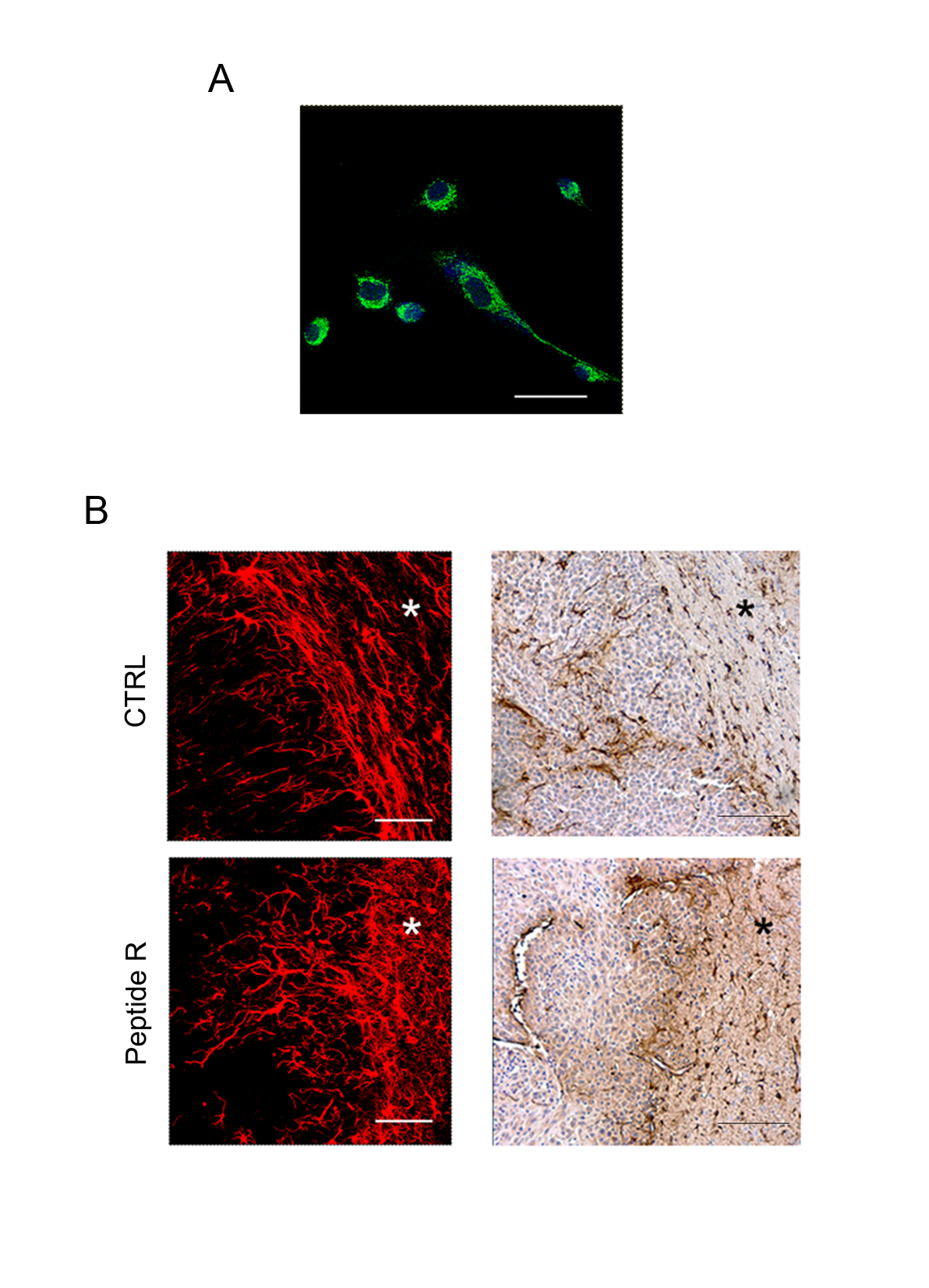

Supplement: Additional file 2: Figure S2. — A) CD68 expression on U87MG cell line. Representative CLSM analysis of CD68 expression (green) on U87MG cells. Nuclei were stained with DAPI (blue). Scale bars, 47 μM. B) In vivo effect of peptide R on astrocytes reactivity. GFAP (marker of astrocyte reactivity) detection by CLSM (on the left) and immunohistochemistry (on the right) on brain sections of mice treated with peptide R or left untreated (CTRL). Asterisks (*) indicate the tumor-free parenchyma. Scale bars, 30 μM. (TIF 1451 kb) [file 13046_2016_326_MOESM2_ESM.tif]
